# Discovery of a Novel Stem Rust Resistance Allele in Durum Wheat that Exhibits Differential Reactions to Ug99 Isolates

**DOI:** 10.1534/g3.117.300209

**Published:** 2017-08-28

**Authors:** Jayaveeramuthu Nirmala, Jyoti Saini, Maria Newcomb, Pablo Olivera, Sam Gale, Daryl Klindworth, Elias Elias, Luther Talbert, Shiaoman Chao, Justin Faris, Steven Xu, Yue Jin, Matthew N. Rouse

**Affiliations:** * Cereal Disease Laboratory, United States Department of Agriculture-Agricultural Research Service, Saint Paul, Minnesota 55108; † Department of Plant Pathology, University of Minnesota, Saint Paul, Minnesota 55108; ‡ Department of Plant Sciences, North Dakota State University, Fargo, North Dakota 58108; § The School of Plant Sciences, University of Arizona, Tucson, Arizona 85721; ** Cereal Crops Research, United States Department of Agriculture-Agricultural Research Service, Fargo, North Dakota 58102; †† Department of Plant Sciences and Plant Pathology, Montana State University, Bozeman, Montana 59717

**Keywords:** Wheat, durum, stem rust, resistance gene, Ug99

## Abstract

Wheat stem rust, caused by *Puccinia graminis* f. sp. *tritici* Eriks. & E. Henn, can incur yield losses in susceptible cultivars of durum wheat, *Triticum turgidum* ssp. *durum* (Desf.) Husnot. Although several durum cultivars possess the stem rust resistance gene *Sr13*, additional genes in durum wheat effective against emerging virulent races have not been described. Durum line 8155-B1 confers resistance against the *P. graminis* f. sp. *tritici* race TTKST, the variant race of the Ug99 race group with additional virulence to wheat stem rust resistance gene *Sr24*. However, 8155-B1 does not confer resistance to the first-described race in the Ug99 race group: TTKSK. We mapped a single gene conferring resistance in 8155-B1 against race TTKST, *Sr8155B1*, to chromosome arm 6AS by utilizing Rusty/8155-B1 and Rusty*2/8155-B1 populations and the 90K Infinium iSelect Custom bead chip supplemented by KASP assays. One marker, *KASP_6AS_IWB10558*, cosegregated with *Sr8155B1* in both populations and correctly predicted *Sr8155B1* presence or absence in 11 durum cultivars tested. We confirmed the presence of *Sr8155B1* in cultivar Mountrail by mapping in the population Choteau/Mountrail. The marker developed in this study could be used to predict the presence of resistance to race TTKST in uncharacterized durum breeding lines, and also to combine *Sr8155B1* with resistance genes effective to Ug99 such as *Sr13*. The map location of *Sr8155B1* cannot rule out the possibility that this gene is an allele at the *Sr8* locus. However, race specificity indicates that *Sr8155B1* is different from the known alleles *Sr8a* and *Sr8b*.

Stem rust caused by *Puccinia graminis* Pers.:Pers. f. sp. *tritici* Eriks. & E. Henn. (*Pgt*) is a devastating fungal disease of both common (*Triticum aestivum* L., 2*n* = 6*x* = 42, AABBDD) and durum wheat (*Triticum turgidum* ssp. *durum* (Desf.) Husnot, 2*n* = 4*x* = 28, AABB), and can culminate in significant yield losses worldwide (Singh *et al.* 2015). This disease has been the culprit behind several famines and epidemics around the world (Bushnell and Roelfs 1984; Kolmer *et al.* 2007; Park 2007). It destroyed 43% of the spring wheat crop, mostly durum, in North Dakota (Peturson 1958) in 1954 and 56% in 1935 (Leonard and Szabo 2005). Since then, this disease has been effectively controlled globally by deploying multiple sources of resistance (Kolmer *et al.* 1991). Near eradication of barberry has also prevented the emergence of virulent races and severe epidemics in the US (Leonard and Szabo 2005). However, the identification of race TTKSK (Ug99) in Africa, which rendered the widely deployed stem rust resistance gene *Sr31* and several other *Sr* genes ineffective, has raised serious concerns (Jin *et al.* 2007; Pretorius *et al.* 2000). This race has spread throughout East Africa, Yemen, Iran, and South Africa (Nazari *et al.* 2009; Jin *et al.* 2008; Pretorius *et al.* 2010; Singh *et al.* 2015; Patpour *et al.* 2016) and is projected to spread further. Race TTKSK has evolved continuously, giving rise to at least 13 different variants, resulting in the defeat of additional stem rust resistance genes (Singh *et al.* 2015; Fetch *et al.* 2016; Newcomb *et al.* 2016). Overall, these new races pose a threat to our food security as 85–95% of wheat cultivars grown around the world are susceptible to at least one of the Ug99 variants (Singh *et al.* 2011).

Race TTKST is a variant of TTKSK (Ug99) that was first discovered in Kenya in 2006. Race TTKST is virulent to both *Sr24* and *Sr31* (Jin *et al.* 2008). In 2007, this race caused severe local epidemics in Kenya. It has since been detected in Tanzania, Ethiopia, Uganda, Eritrea, Rwanda, and Egypt (Jin *et al.* 2008; Pretorius *et al.* 2010; Wolday *et al.* 2011; Hale *et al.* 2013) and was the most prevalent *Pgt* race in Kenya from 2007 to 2014 (Newcomb *et al.* 2016). Races TTTSK, TTKTK, and TTKTT with additional virulences to *Sr24*, *Sr36*, and *SrTmp* (Jin *et al.* 2009; Newcomb *et al.* 2016) have substantially increased the vulnerability of wheat to stem rust because of the widespread use of these genes in global wheat breeding.

There is an urgent need to find additional genes that confer resistance to the new races of the Ug99 race group and identify reliable markers that assist breeding programs in combining these genes in desirable germplasm. However, genetic diversity for stem rust resistance in conventional common and durum wheat gene pools is limited (Singh *et al.* 2011), which has been a major constraint in identifying new genes effective against the Ug99 race group. Tetraploid wheats (*T. turgidum* ssp.) have contributed stem rust resistance genes such as *Sr2*, *Sr9d*, *Sr9e*, *Sr9g*, *Sr11*, *Sr12*, *Sr13*, *Sr14*, and *Sr17* (McIntosh 1988; Simons *et al.* 2011; Singh *et al.* 2006, 2011). However, *Sr13* is the only known Ug99-effective seedling *Sr* gene present in selected durum cultivars in the US (Simons *et al.* 2011). Screening wheat lines for seedling resistance against race TTKST led to the identification of a durum line called 8155-B1 that was resistant to race TTKST, but susceptible to race TTKSK. This was the first line known to possess resistance to a variant of TTKSK that is considered to be more virulent than TTKSK. 8155-B1 was also resistant to US race TMLKC and exhibited a phenotype similar to TTKST. However, the basis of this resistance was unknown. The goal of this research was to determine the genetic basis of resistance to races TTKST and TMLKC in the durum line 8155-B1 and to develop KASP (Kompetitive Allele Specific PCR) assay-based SNP markers that can be used to postulate the presence of this gene in uncharacterized germplasm.

## Materials and Methods

### Plant materials

8155-B1 is a *T. turgidum* ssp. *durum* line developed by Norman Williams (USDA-ARS, Fargo, ND) with the pedigree Marruecos 9623//Marruecos 9623/CItr 8155 that was characterized and selected as monogenic for stem rust resistance derived from CItr 8155 (Williams and Gough 1968). Marruecos 9623 (PI 192334) is a stem rust susceptible durum cultivar from Morocco (Williams and Gough 1968). CItr 8155 is a selection of wheat accession PI 59284 that was collected from Ethiopia in 1924. Rusty is a stem rust susceptible durum wheat line (Klindworth *et al.* 2006). Out of 143 Rusty*2/8155-B1 BC_1_F_2_ families, 44 lines that were either susceptible or segregating to race TTKST were employed to initially map the TTKST resistance. Similarly, out of a total of 473 F_2_ plants derived from Rusty/8155-B1, 152 F_2_ plants that were clearly resistant or susceptible to race TMLKC (see *Results* section) were used to map the TMLKC resistance utilizing the 90K Infinium iSelect Custom bead chip SNP genotyping platform. KASP assay-based markers were developed and evaluated on all the 143 BC_1_F_2_ families and on a set of 11 durum cultivars from the US. Eleven durum cultivars and five bread wheat genetic stock lines were evaluated with *Pgt* at the seedling stage and KASP assays to validate mapping results (Supplemental Material, Table S1). Previously, durum cultivar Mountrail (Elias and Miller 2000) was crossed to common wheat variety Choteau (Lanning *et al.* 2004) and two recombinant inbred line (RIL) populations at both 4× and 6× ploidy were derived composed of 96 and 123 individuals, respectively (Kalous *et al.* 2015). We assessed the seedling response of these populations to races TTKSK and TTKST in order to map the response to race TTKST using the previously constructed linkage map with SNPs genotyped utilizing the 90K Infinium iSelect Custom bead chip (Kalous *et al.* 2015).

### Stem rust assays

A total of 473 F_2_ plants along with 8155-B1 and Rusty were evaluated against *Pgt* race TMLKC (isolate 72-41-Sp2) using a method described by Williams *et al.* (1992) at the USDA-ARS Cereals Crops Research Unit, Fargo, ND. At a biocontainment facility at the University of Minnesota, 25 BC_1_F_2_ plants from each of the 143 families were evaluated against *Pgt* race TTKST (isolate 06KEN19v3) along with Rusty and 8155-B1. Choteau, Mountrail, and the 4× and 6× Choteau/Mountrail populations were assessed in two biological replicates each for response to races TTKST and TTKSK (Ug99; 04KEN156/04). Inoculation of seedlings was performed according to previously described methods (Rouse *et al.* 2011). The 11 durum cultivars in addition to 8155-B1 and Rusty were evaluated with TTKSK and its variants, namely TTKST, TTTSK (07KEN24-4), TTKTT (14KEN58-1), and TTKSF+ (09ZIM01-2; race TTKSF with additional virulence to *Sr9h* [Pretorius *et al.* 2012; Rouse *et al.* 2014]). Races TTKSK, TTKST, TTTSK, TTKTT, and TTKSF+ are all members of the Ug99 race group (Newcomb *et al.* 2016). This panel was also evaluated with races JRCQC (08ETH03-1) and TRTTF (06YEM34-1) at both high (22/25° night/d) and low temperatures (15/18° night/d) with a 16-hr photoperiod in growth chambers. These two races were reported from Yemen and Ethiopia and described as particularly virulent to durum wheat (Olivera *et al.* 2012). The letters of each race name correspond to reaction patterns of the isolate to four stem rust resistance genes each (Jin *et al.* 2008). The full avirulence/virulence formulae for the isolates used this study are listed in Table S2.

Seedling evaluations of the biparental populations were conducted in greenhouse conditions (Rouse *et al.* 2011) and infection types were determined 14 d after inoculation following the 0–4 scale developed by Stakman *et al.* (1962). There are six categories of infection types in this rating scale: infection type “0” indicates an immune response with no visible symptoms, “;” indicates chlorotic or necrotic hypersensitive reactions without sporulation, “1” indicates small round rust pustules surrounded by chlorosis or necrosis, “2” indicates rust pustules surrounded by “green islands” of host tissue that are surrounded by chlorosis, “3” indicates elongated (not round) rust pustules, and “4” represents large elongated rust pustules without the presence of chlorosis or necrosis. Variation within each infection type class was captured by the use of “+” and “−” symbols that indicate relatively larger or smaller rust pustule sizes, respectively. When multiple infection types were observed on the same leaf, all infection types were listed, with the most common infection type listed first. A “/” symbol was used to separate multiple infection types recorded for a heterogeneous line where different plants within the same line displayed different infection types. Infection types “0” to “2” were classified as “low,” *i.e.*, incompatible interactions indicative of host resistance and pathogen avirulence, whereas infection types “3” and “4” were classified as “high,” *i.e.*, compatible interactions indicative of host susceptibility. Two biological replicates of seedling screening of the cultivar panel were performed. Significant deviation from the expected Mendelian genotypic frequencies was tested using chi-square tests.

### SNP genotyping and identification of markers linked to race TTKST resistance

DNA from 22 susceptible and 22 segregating BC_1_F_2_ families in response to race TTKST and 152 F_2_ plants segregating for response to race TMLKC were isolated using a modified CTAB extraction method (Rouse *et al.* 2012) or an ammonium acetate method (Pallotta *et al.* 2003) and resuspended in water. Tissue from 10 BC_1_F_2_ plants from each family was bulked for extraction of DNA representing each BC_1_F_2_ family. DNA isolated from these lines was genotyped at the USDA-ARS Cereal Crops Research Unit, Fargo, ND, with 90,000 gene-based SNPs using a custom Infinium iSelect bead chip array and an iScan following the manufacturer’s instructions (Illumina Inc., Hayward, CA; Wang *et al.* 2014). Allele calls were performed using the genotyping module of GenomeStudio v2011.1 software (Illumina Inc.) for the BC_1_F_2_ population, whereas the polyploidy clustering module of the software was used to score the alleles of the F_2_ population. The SNP consensus map data (Wang *et al.* 2014) were imported into GenomeStudio software to assign chromosome positions.

### Identification of linked markers and map construction

Pearson correlations were used initially to identify markers associated with the TTKST phenotype in the subset of 44 families belonging to the BC_1_F_2_ population (*t*-tests with *P* < 0.05). The top 16 SNP markers (*P* < 0.03) significantly correlated with resistance to race TTKST were converted into KASP assays. Eight of these KASP assays were polymorphic and were evaluated on the 143 BC_1_F_2_ lines. These data were used to generate a linkage map using JoinMap version 4.0 (Stam 1993; Van Ooijen 2006). Genetic distances were calculated using the Kosambi mapping function (Kosambi 1944), and linkage groups were formed at logarithm of odds (LOD) value of 5.0 and 40% maximum recombination frequency. The KASP assay-based markers were also screened on the 11 cultivars. The primer sequences designed for the KASP assays are provided in Table 1. For the F_2_ population, mapping of resistance to TMLKC was performed by calculating a linkage map using MapDisto 1.7.7 (Lorieux 2012). Linkage groups were created using the LOD score and Rmax value of 3.0. Map distances were calculated using the Kosambi mapping function (Kosambi 1944). For the Choteau/Mountrail populations, hexaploid and tetraploid lines were combined into one population for the purpose of mapping. Previously available 90K SNP data (Kalous *et al.* 2015) were combined with binary resistance data in order to map the loci corresponding to resistance to races TTKSK and TTKST.

**Table 1:** Primers used for KASP assays for markers derived from the 90K iSelect assay on chromosome arm 6AS

KASP Primer	Primer Type*^a^*	Primer Sequence
*KASP_6AS_IWB72958*	A1	GAAGGTGACCAAGTTCATGCTGGCTGCTGCCAACTCCCCA
	A2	GAAGGTCGGAGTCAACGGATTGCTGCTGCCAACTCCCCG
	C1	GTACTGTGAGTGTCTCGGATGTTGAT
*KASP_6AS_IWB64918*	A1	GAAGGTGACCAAGTTCATGCTGCACTTGCGACTCGAGGGTT
	A2	GAAGGTCGGAGTCAACGGATTGCACTTGCGACTCGAGGGTC
	C1	GGCCCGGAATCCGCCACCAT
*KASP_6AS_IWB12224*	A1	GAAGGTGACCAAGTTCATGCTGTTCTGCGTTGGAAATAATTTCTAGG
	A2	GAAGGTCGGAGTCAACGGATTCGTTCTGCGTTGGAAATAATTTCTAGT
	C1	GACTTATCATGTGCTCATCAGGTTAAGTT
*KASP_6AS_IWB75264*	A1	GAAGGTGACCAAGTTCATGCTAACGTGCACATCGCTTACCGC
	A2	GAAGGTCGGAGTCAACGGATTGAACGTGCACATCGCTTACCGT
	C1	GGCCGTCGGGAACTCCACAAA
*KASP_6AS_IWB43809*	A1	GAAGGTGACCAAGTTCATGCTGCTGCCAACTCCCCG
	A2	GAAGGTCGGAGTCAACGGATTGGCTGCTGCCAACTCCCCA
	C1	GTACTGTGAGTGTCTCGGATGTTGAT
*KASP_6AS_IWB1550*	A1	GAAGGTGACCAAGTTCATGCTAAAGGTGAAAGGAGCTGTTCACAGT
	A2	GAAGGTCGGAGTCAACGGATTGGTGAAAGGAGCTGTTCACAGC
	C1	TCTGTCCTTCTCTGTCCTGGCAAT
*KASP_6AS_IWB61585*	A1	GAAGGTGACCAAGTTCATGCTCCGTCAGAGAGATCATCAGAGG
	A2	GAAGGTCGGAGTCAACGGATTACCGTCAGAGAGATCATCAGAGA
	C1	TATCTCATCACAAGTTGAGCATACTCAGA
*KASP_6AS_IWB10558*	A1	GAAGGTGACCAAGTTCATGCTGATGGTTGTATACGGGCCTATGG
	A2	GAAGGTCGGAGTCAACGGATTGATGGTTGTATACGGGCCTATGA
	C1	CTCAGCTGGCATGTATTTTTGGGGAT

a Primer types A1 and A2 are allele-specific primers, whereas primer type C1 is a common primer for both alleles.

### KASP reaction conditions

Each KASP PCR consisted of 50 ng of DNA template, 5 µl of 2× KASP buffer, and 0.14 µl of primer mixture. Thermal cycling conditions were 94° for 15 min, followed by 10 cycles of touch down PCR: 94° for 20 sec, 65–57° for 60 sec (dropping 0.8° per cycle), followed by 36 cycles of regular PCR: 94° for 20 sec, 57° for 60 sec, followed by fluorescence reading at 20°. A total of 3–9 additional cycles of PCR were added to obtain a good separation of clusters, as needed. Both thermal cycling and fluorescence reading were performed on an ABI StepOnePlus Real-Time PCR system. At least two replicates of each KASP assay were performed. If inconsistent results were observed between the two replicates, a third replicate was performed.

### Data availability

All data that we used to draw conclusions in this article are represented either within the article, or in the Supplemental Material. Table S1 describes the wheat lines used in this study. Table S2 describes the *Pgt* isolates used in this study. Table S3 describes the number of Rusty/8155-B1 F_2_ progeny with specific infection types observed in response to *Pgt* race TMLKC. Table S4 lists 90K SNP markers identified as correlated with response to *Pgt* race TTKST in a selection of the BC_1_F_2_ population. Table S5 contains the alleles of markers linked to *Sr8155B1* in Rusty*2/8155-B1 families. Table S6 contains the alleles of markers linked to *Sr8155B1* in Choteau/Mountrail RILs that displayed recombination events near *Sr8155B1*. Table S7 contains alleles of markers mapped to chromosome 6A in the Rusty/8155-B1 F_2_ population. Table S8 contains seedling infection types observed on F_2_ plants from the Rusty/8155-B1 population. Table S9 contains seedling infection types observed on BC_1_F_2_ families of Rusty*2/8155-B1. Table S10 contains seedling infection types observed on Choteau/Mountrail RILs in response to races TTKST and TTKSK. Figure S1 displays a range of seedling infection types observed on Rusty/8155-B1 F_2_ progeny in response to *Pgt* race TMLKC. Figure S2 displays the genetic linkage map derived from Rusty/8155-B1 F_2_ progeny. Figure S3 displays the seedling infection types observed on Choteau and Mountrail in response to *Pgt* race TTKST.

## Results

### Genetic basis of resistance of 8155-B1 to Pgt races TTKST and TMLKC

**Figure 1:**
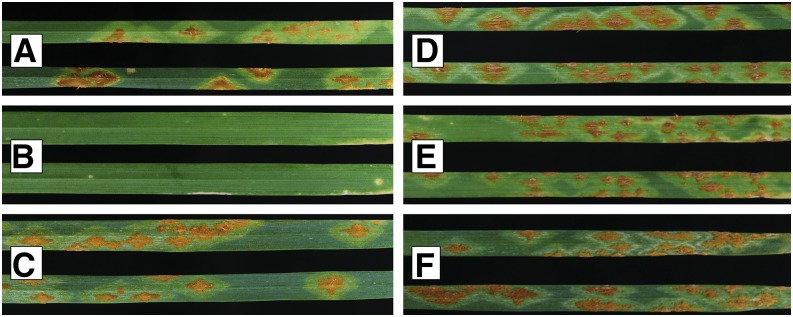
Race specificity of seedling infection types exhibited by durum line 8155-B1 in response to *Puccinia graminis* f. sp. *tritici* races TTKST and TTKSK. (A–C) are lines that were inoculated with race TTKST, whereas (D–F) are lines inoculated with race TTKSK. (A and D) correspond to wheat line Rusty, (B and E) to 8155-B1, and (C and F) to Desert King HP.

The durum line 8155-B1 exhibited an infection type of “0;” to “0;1,” whereas Rusty exhibited an infection type of “3+” to race TTKST (Figure 1) at both high and low temperature regimes. Race TMLKC manifested an infection type of “0;” on 8155-B1 and “3+” to “4” on Rusty (Figure S1). The F_2_ progeny segregated for resistance to race TMLKC, with 112 plants displaying infection types characteristic of 8155-B1-type resistance (“0;”, “;”, “;1−”, “;1”, “12”, “2;”, and “23;”) and 361 plants not exhibiting this type of resistance (infection types “2”, “23”, “32”, “3”, “43”, and “4”) (Table S3). The presence of hypersensitive reactions “;” and “1” were considered indicative of the presence of resistance derived from 8155-B1. Segregation for resistance among the F_2_ plants did not deviate from the expected ratio for a single recessive gene (*χ*^2^ = 0.44, *P* = 0.51). Out of the 75 F_2_ plants with infection type “0;” to “;”, 72 were selected for genotyping. Out of the 84 F_2_ plants with infection type “4,” 80 with adequate DNA extractions were used for genotyping.

The BC_1_F_2_ families exhibited infection types (“23−” to “4”) to race TTKST when homozygous, and segregated for resistant infection types (“0;1” to “;13−”) and susceptible infection types (“23−” to “4”) when heterozygous. The segregation of resistance within BC_1_F_2_ families classified as heterozygous (865 total plants) did not deviate from the expected ratio for a single recessive gene (*χ*^2^ = 0.086, *P* = 0.79). The ratio of homozygous *vs.* heterozygous families deviated from the expected 1:1 ratio with *χ*^2^ = 16.79 (*P* = 4.2 × 10^−5^). The linked KASP assay-based SNP markers also deviated from the expected 1:1 ratio (Table 2), indicating that there was segregation distortion at this locus in the BC_1_F_2_ population. The resistance gene was tentatively designated as *Sr8155B1*.

**Table 2:** Segregation distortion for response to *Puccinia graminis* f. sp. *tritici* race TTKST and closely linked KASP markers among Rusty*2/8155-B1 BC_1_F_2_ families

	Homozygous			
Marker	Rusty allele	Heterozygous	*χ^2^*(1:1)	*P*
TTKST response	96	47	16.79	4.2 × 10^−5^
*KASP_6AS_IWB64918*	87	46	12.64	3.8 × 10^−4^
*KASP_6AS_IWB1550*	93	47	15.11	1.0 × 10^−4^
*KASP_6AS_IWB72598*	89	52	9.71	0.002
*KASP_6AS_IWB43809*	91	50	11.92	5.5 × 10^−4^
*KASP_6AS_IWB75264*	86	50	9.53	0.0002
*KASP_6AS_IWB10558*	95	43	19.59	9.6 × 10^−6^
*KASP_6AS_IWB61585*	93	46	15.89	6.7 × 10^−5^
*KASP_6AS_IWB12224*	86	46	12.12	5.0 × 10^−4^

### Molecular mapping of Sr8155B1

**Figure 2:**
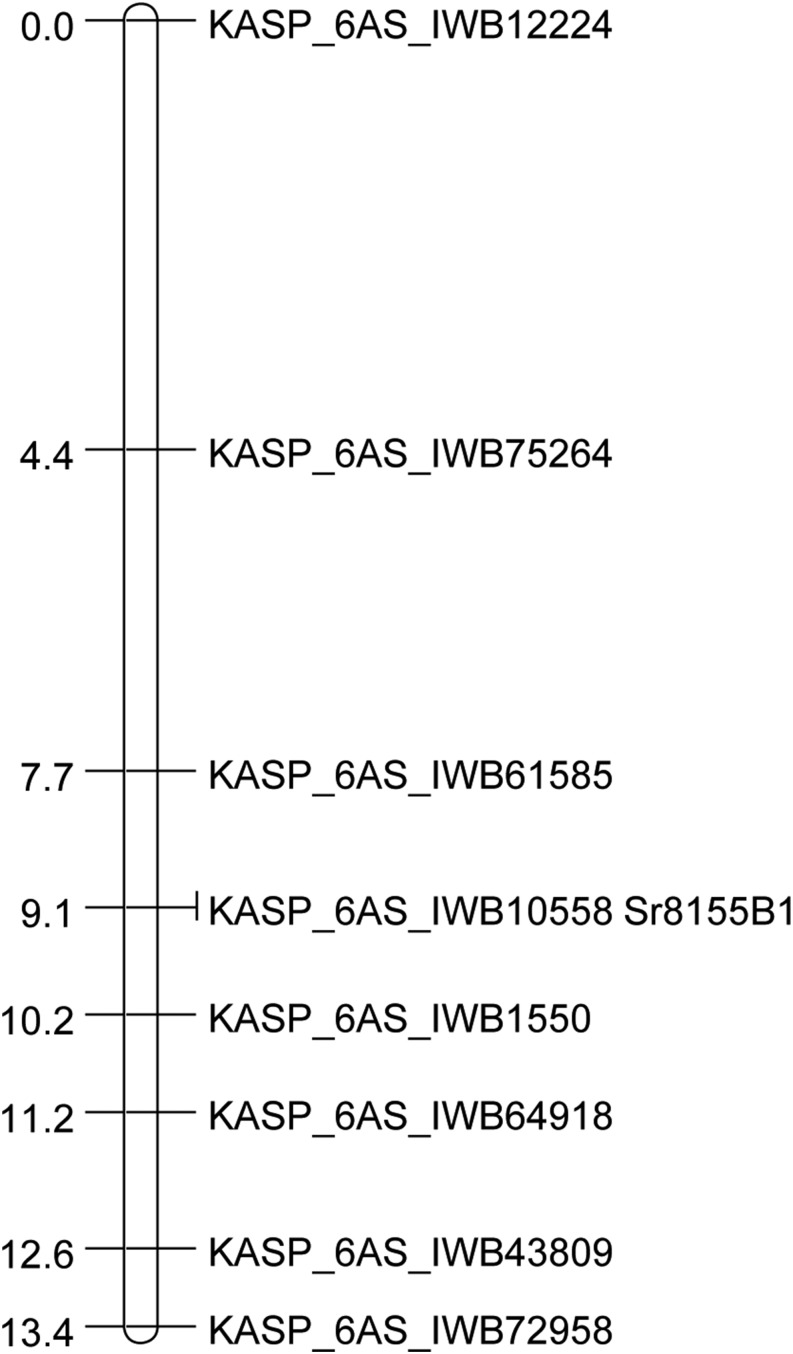
Genetic linkage map including *Sr8155B1* and KASP assay SNP markers on chromosome arm 6AS constructed from Rusty*2/8155-B1 BC_1_F_2_ progeny. The values to the left of the marker names are the distances (cM) generated using the Kosambi distance function.

From a total of 21 SNPs significantly correlated with race TTKST resistance in the 44 BC_1_F_2_ families (Table S4), eight polymorphic KASP assay-based markers were evaluated on 143 BC_1_F_2_ families (Table 1). Seven markers were linked to the TTKST-resistant phenotype, including KASP_6AS_IWB10558 that cosegregated with *Sr8155B1* (Figure 2 and Table S5). Markers KASP_6AS_IWB61585 and KASP_6AS_IWB1550 flanked *Sr8155B1* at 1.3 cM distal and 1.1 cM proximal, respectively. From the 152 F_2_ plants, we generated a linkage map of 116 cM (Figure S2). The linkage map clearly positioned *Sr8155B1* in the short arm of chromosome 6A, and was tightly linked to three markers that also were linked to *Sr8155B1* in the BC_1_F_2_ population: *IWB64918*, *IWB43809*, and *IWB10558*. *Sr8155B1* was flanked by the SNP markers *IWB55188* and *IWB35219*, being 7.3 cM proximal and 0.7 cM distal to *Sr8155B1*, respectively. The corresponding positions of these markers in the durum consensus map (Maccaferri *et al.* 2014) are shown in Table S4. Previously, wheat stem rust resistance gene *Sr8*, including alleles *Sr8a* and *Sr8b*, was mapped to the short arm of chromosome 6A (McIntosh 1972; Singh and McIntosh 1986; Bhavani *et al.* 2008).

### Postulation of Sr8155B1 and Sr13 in durum wheat cultivars

The 11 durum cultivars displayed a range of infection types in response to races TTKST, TTKTT, TTTSK, TTKSF+, TTKSK, TRTTF, and JRCQC (Table 3). A low infection type of “0;” to “;1” in response to race TTKST in combination with a higher infection type in response to race TTKSK was considered indicative of resistance conferred by *Sr8155B1*. Of the 11 cultivars evaluated, nine were postulated to possess *Sr8155B1* (Table 3). The nine cultivars with *Sr8155B1* in addition to 8155-B1 exhibited consistently low infection types at both temperature regimes to races TTKTT, TTTSK, TTKSF+, and TRTTF (Table 3), but not necessarily to race JRCQC. A low infection type of “11+” to “22+” in response to race TTKSK at the higher temperature regime was considered indicative of resistance conferred by *Sr13* (Roelfs and McVey 1979). Six cultivars were postulated to possess *Sr13* (Table 3). Five cultivars were postulated to possess both *Sr13* and *Sr8155B1*: Rugby, Munich, Renville, Grenora, and Alkabo.

**Table 3:** Seedling infection types of 11 US durum cultivars in addition to 8155-B1 and Rusty in response to *Puccinia graminis* f. sp. *tritici* races TTKSK, TTKST, TKTTF, TTTSK, TTKSF+, TRTTF, and JRCQC at low and high temperature regimes

			18/15° day/night							25/22° day/night						
Line	*Sr8155B1**^a^*	*Sr13**^b^*	TTKSK	TTKST	TTKTT	TTTSK	TTKSF+	TRTTF	JRCQC	TTKSK	TTKST	TTKTT	TTTSK	TTKSF+	TRTTF	JRCQC
D.K. HP*^c^*	—	—	3+4*^d^*	3+	—	3+	3+	3+	3+	3+	3+4	—	3+	3+	3+	3+
Rugby	+	+	2+3	;1−	0;	0;1−	0;	0;	23−	2−	0;12−	0;	0;	0;/2−	0;	32+
Dilse	+	—	3+	0;	0;	0;	0;	0;	3+	3+	0;	0;	0;	0;	0;	33+
Munich	+	+	1+3	0;	0;	0;	0;	0;	33+	11+	0;	0;	0;	0;	0;	33+
Renville	+	+	33+	0;	0;	0;	0;	0;	3	2	0;	0;	0;	0;	0;	2
Belzer	+	—	3+	0;	0;	0;	0;	0;	3+	3+	0;	0;	0;	0;	0;	3+
Grenora	+	+	32+	0;	0;	0;	0;	0;	3	22+	0;	0;	0;	0;	0;	32+
Lloyd	+	—	3+	0;	0;1	;1	;1/0;	0;	3+	3+	0;	0;1	0;	0;	0;	3+
Divide	+	—	3+	0;1	0;	0;1	0;	0;	3+	3+	0;	0;	0;	0;	0;	3+
Tioga	—	+	33+	23−	22+	23−	12−	12−	3+	22−	12−	12−	2−	12−	1	3+
Alkabo	+	+	3	0;	0;	0;	0;	0;	33−	22+	0;	0;	0;	0;	0;	33+
8155-B1	+	—	3+	;	0;	;	0;	0;	3+	3+	0;1	0;	0;1	0;1	0;	3+
Rusty	—	—	3+	3+	3+	3+	3+	3+	3+	3+	3+	3+	3+	3+	3+	3+

a *Sr8155B1* postulations were made based on low infection type in response to race TTKST, and higher infection type observed in response to race TTKSK.

b *Sr13* postulations were made based on the presence of an intermediate infection type in response to virulent race TTKSK at the higher temperature regime at which *Sr13* is most effective.

c Desert King HP.

d Stakman *et al.* (1962) infection types on a “0” to “4” scale are listed, where “0” indicates immunity, “4” indicates compete susceptibility, and “;” indicates a class in between “0” and “1” (see *Materials and Methods* for a detailed description). Smaller or larger rust pustules within an infection type class are denoted by “−” and “+” symbols, respectively. When multiple infection types were observed on the same leaf, all infection types are listed. A “/” symbol was used to separate infection types observed on different plants of the same heterogeneous line.

### Validation of Sr8155B1-linked markers in durum cultivars

To identify potential markers that can discriminate lines with and without *Sr8155B1*, we evaluated eight KASP assay-based markers on the panel of 11 durum cultivars. The allele calls for these eight markers are shown in Table 4. Of the eight markers tested, three markers were found to predict the presence of *Sr8155B1*: *KASP_6AS_IWB10558* (cosegregated with *Sr8155B1*), *KASP_6AS_IWB72958*, and *KASP_6AS_IWB61585* (Table 4).

**Table 4:** Allele calls of eight KASP assay SNP markers linked to *Sr8155B1*

Lines	*Sr8155B1**^a^*	1	2	3	4	5*^b^*	6	7	8
Desert King HP	—	a	a	a	a	b	b	b	b
Rugby	+	a	a	a	a	a	a	b	a
Dilse	+	a	a	a	a	a	a	b	a
Munich	+	a	—	h	a	a	a	b	a
Renville	+	a	a	a	a	a	a	a	a
Belzer	+	a	a	a	a	a	a	b	a
Grenora	+	a	a	a	a	a	a	b	a
Lloyd	+	a	a	a	a	a	a	b	a
Divide	+	a	a	a	a	a	a	b	a
Tioga	—	b	h	b	b	b	—	b	b
Alkabo	+	a	b	a	a	a	a	b	a
8155-B1	+	a	a	a	a	a	a	a	a
Rusty	—	b	b	b	b	b	b	b	b

1: *KASP_6AS_IWB12224*, 2: *KASP_6AS_IWB64918*, 3: *KASP_6AS_IWB_43809*, 4: *KASP_6AS_IWB75264*, 5: *KASP_6AS_IWB10558*, 6: *KASP_6AS_IWB72958*, 7: *KASP_6AS_IWB1550* and 8: *KASP_6AS_IWB61585*.

a *Sr8155B1* gene postulated based on phenotypic data.

b Closest linked marker to *Sr8155B1*.

### Relationship between Sr8155B1 and the Sr8 alleles

**Figure 3:**
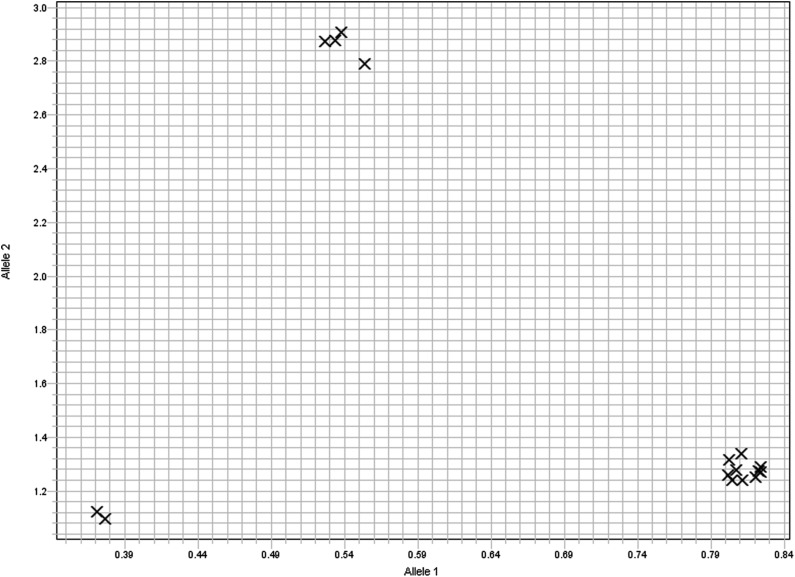
Allelic discrimination plot showing the SNP marker *KASP_6AS_IWB10558* alleles. The *Y*-axis (allele 2) depicts amplification of the resistant-linked allele represented by lines 8155-B1 and Divide, whereas the *X*-axis (allele 1) represents amplification of the susceptible-linked allele represented by lines Rusty, Barletta Benvenuota (*Sr8b*), Klein Titan (*Sr8b*), IsSr8a (*Sr8a*), and SD4279 (*Sr8a*). Duplicate reactions are displayed for all the lines described in the figure. Duplicate water negative controls displayed null reactions with no amplification of either allele.

The marker *KASP_6AS_IWB10558* was also evaluated on the *Sr8a*-containing lines ISr8a-Ra and SD4279 and the *Sr8b*-containing lines Barletta Benvenuota and Klein Titan, along with a highly susceptible wheat line, LMPG-6. The results revealed that ISr8a-Ra, SD4279, Barletta Benvenuota, Klein Titan, and LMPG-6 did not have the 8155-B1 allele for *KASP_6AS_IWB10558* (Figure 3 and Table 5). ISr8a-Ra is susceptible to races TTKSK and TTKST, but resistant to TRTTF (Olivera *et al.* 2012), whereas *Sr8b* lines Klein Titan and Barletta Benvenuota were susceptible to races TTKSK, TTKST, and TRTTF (Table 5). Although 8155-B1 is resistant to TRTTF, it differs from ISr8a-Ra in that it produces an IT of “0;” whereas ISr8a-Ra exhibits an infection type “22−”(Table 5). The race specificity and infection types of ISr8a-Ra, Klein Titan, and Barletta Benvenuota clearly indicate that *Sr8155B1* is different from both *Sr8a* and *Sr8b*.

**Table 5:** Seedling infection types exhibited by lines with *Sr8a*, *Sr8b*, and *Sr8155B1* in response to *Puccinia graminis* f. sp. *tritici* races TRTTF, TTKSK, and TTKST

Race	G*ene*	TRTTF	TTKSK	TTKST
8155-B1	*Sr8155B1*	0;*^a^*	3+	0;
ISr8a-Ra	*Sr8a*	22−	4	3+
SD4279	*Sr8a + Sr9h*	2	22+	22+
Klein Titan	*Sr8b*	4	4	4
Barletta Benvenuota	*Sr8b*	4	4	4
LMPG-6	—	3+	3+	3+
Rusty	—	3+	4	3+

a Stakman *et al.* (1962) infection types on a “0” to “4” scale are listed, where “0” indicates immunity, “4” indicates compete susceptibility, and “;” indicates a class in between “0” and “1” (see *Materials and Methods* for a detailed description). Smaller or larger rust pustules within an infection type class are denoted by “−” and “+” symbols, respectively. When multiple infection types were observed on the same leaf, all infection types are listed.

### Validation of the presence of Sr8155B1 in cultivar Mountrail

Choteau displayed susceptible infection type “3+” in response to races TTKSK and TTKST. Mountrail displayed infection type “2−” in response to race TTKSK, and infection types “0;” to “;” in response to race TTKST (Figure S3). The response of Mountrail to races TTKSK and TTKST is typical of cultivars postulated to possess both *Sr13* and *Sr8155B1* (Table 3). The RILs derived from Choteau/Mountrail displayed two classes of infection types in response to race TTKSK: a resistant class with a range between “;12−” and “2”, in addition to a susceptible class with a range between “3” and “3+”. Segregation of response to race TTKSK fit a single gene (*χ*^2^ = 1.73, *P* = 0.19), likely *Sr13*. The response of the RILs to race TTKST included infection types ranging from “0;” to “3+”. Segregation of resistance to race TTKST fit a two-gene model (*χ*^2^ = 1.91, *P* = 0.17), likely *Sr13* and *Sr8155B1*. We classified RILs with resistant TTKST infection types that displayed lower infection types compared to the response to race TTKSK as possessing *Sr8155B1* (Table S10). RILs with TTKST infection types that displayed similar infection types to race TTKSK were classified as lacking *Sr8155B1* (Table S10). We postulated that 91 RILs possessed *Sr8155B1* and 108 lacked the gene, which fit a single gene ratio (*χ*^2^ = 1.45, *P* = 0.23). We were not able to confidently postulate presence or absence of *Sr8155B1* in 20 of the 219 RILs (Table S10). We did observe a small quantitative difference between the effect of *Sr8155B1* in hexaploid and tetraploid RILs of the Choteau/Mountrail population. The four most common infection types of hexaploid RILs (SXD1 through SXD135) postulated to possess *Sr8155B1* were “;1−”, “;1”, “11+”, and “;13−”, but the most common infection types for tetraploid RILs (SXD136 to SXD232) with *Sr8155B1* were “0;”, “;”, “;1−”, and “;1” (Table S10).

**Figure 4:**
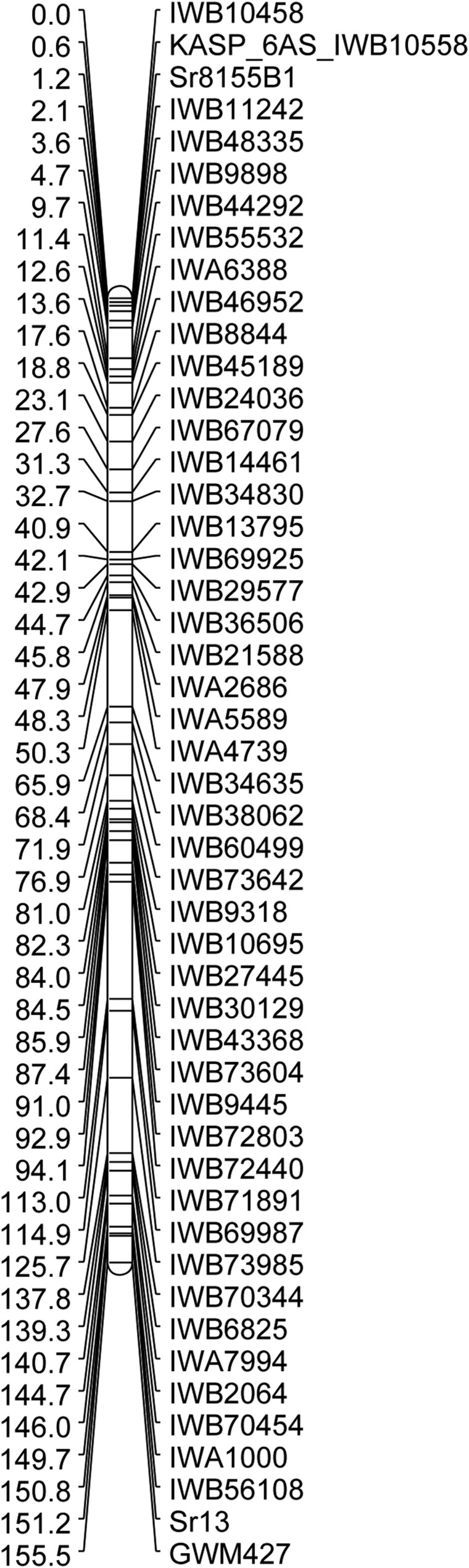
Genetic linkage map of chromosome 6A including *Sr8155B1* and *Sr13* using 90K SNP markers and *KASP_6AS_IWB10558* from Choteau/Mountrail recombinant inbred lines. The values to the left of the marker names are the distances (cM) generated using the Kosambi mapping function.

Both *Sr13* and *Sr8155B1* were mapped in the Choteau/Mountrail population to chromosome 6A (Figure 4). *Sr8155B1* mapped on the distal end of chromosome arm 6AS, whereas *Sr13* mapped 150.6 cM away on the distal end of chromosome arm 6AL. Previously, *Sr13* was linked to *GWM427* in multiple populations (Simons *et al.* 2010). In Choteau/Mountrail, *Sr13* mapped 4.3 cM proximal to *GWM427*. *Sr8155B1* mapped 0.6 cM proximal to *KASP_6AS_IWB10558*. Markers *KASP_6AS_IWB1550* and *KASP_6AS_IWB61585* were not polymorphic in the Choteau/Mountrail population. Only two RILs possessed recombination events between *Sr8155B1* and *KASP_6AS_IWB10558* (SXD100 and SXD128; Table S6), demonstrating their tight linkage and the tractability of *KASP_6AS_IWB10558* in another population. These mapping results confirm the presence of both *Sr8155B1* and *Sr13* in the cultivar Mountrail.

## Discussion

The search for new effective stem rust resistance genes from diverse germplasm is a continuous process in breeding wheat for resistance to stem rust. The best strategy to control emerging virulent races is to deploy complex rust resistance by adding new genes from diverse sources into a highly durable genetic background (Park *et al.* 2007, 2008). Monogenic line 8155-B1 line is unique because it is resistant to race TTKST, a variant of TTKSK with increased virulence, but susceptible to race TTKSK. 8155-B1 is also resistant to other variants in the Ug99 race group including TTTSK, TTKSF+, and TTKTT. In addition, 8155-B1 is resistant to TMLKC and TRTTF. Avirulence to *Sr8155B1* is found in isolates of the Ug99 race group that vary in their avirulence to resistance genes *Sr9h*, *Sr24*, *Sr31*, *Sr36*, and *SrTmp*, and only one isolate has been characterized as virulent to *Sr8155B1* (04KEN156/04; TTKSK). Although 04KEN156/04 is the oldest isolate in the Ug99 race group that we tested, we expect that ancestral Ug99 race group isolates are likely avirulent to *Sr8155B1* based on the geographic and genetic variability of isolates that we confirmed as avirulent to *Sr8155B1* (Newcomb *et al.* 2016). We hypothesize that virulence to *Sr8155B1* is likely conferred by loss or modification of dominant avirulence to *Sr8155B1*. *Pgt* avirulence to most wheat stem rust resistance genes tested segregated as single dominant genes (Zambino *et al.* 2000). *Melampsora lini* avirulence proteins characterized in the flax-flax rust pathosystem directly interacted with flax resistance genes to induce host resistance (Dodds *et al.* 2006). We expect *Sr8155B1*-mediated resistance to similarly be conferred by the presence of a unique resistance-avirulence protein pair. Another possibility is that *Sr8155B1* virulence is ancestral in the Ug99 race group, meaning that *Sr8155B1* avirulence was acquired. This scenario might be explained by a mutation event that is either coincidental with the diversification of the Ug99 race group or associated with a selective advantage conferred by *Sr8155B1* avirulence. If avirulence to *Sr8155B1* was acquired, the molecular interactions causing this would be valuable to dissect to improve our understanding of such a phenomenon. Testing multiple isolates of each race for reaction to *Sr8155B1* would help elucidate the evolution of virulence to *Sr8155B1* in the Ug99 race group.

Segregation of resistance based on genetic analyses of F_2_ and BC_1_F_2_ populations revealed that a single gene, *Sr8155B1*, conferred resistance to race TTKST. Segregation of resistance fit the inheritance of a recessive gene in both populations, but it was difficult to determine whether *Sr8155B1* is truly recessive or incompletely dominant (Williams and Gough 1968) without careful testing of F_1_ plants derived from Rusty/8155-B1. Testing of 8155-B1 and several *Sr8155B1*-possessing cultivars at two temperature regimes indicated that *Sr8155B1* is stable at high and low temperatures, in contrast to *Sr13* and *Sr21* (Table 3; Chen *et al.* 2015). Testing hexaploid and tetraploid RILs of the Choteau/Mountrail population indicated that *Sr8155B1* was more effective in tetraploid wheat, similar to findings for other *Sr* genes including *Sr13* and *Sr21* (Chen *et al.* 2015; Simons *et al.* 2010). Resistance to races TTKST and TMLKC mapped to the short arm of chromosome 6A and cosegregated with SNP marker *IWB10558* in both the F_2_ and BC_1_F_2_ progeny, suggesting that the same gene conditions resistance against both races. The only other characterized gene from durum wheat that confers seedling resistance to race TTKST is *Sr13*, which is effective against all known races in the Ug99 race group and was mapped to the long arm of chromosome 6A (McIntosh 1972; Simons *et al.* 2011). To date, no known gene(s) on chromosome 6AS have been described to confer seedling resistance against race TTKST. However, the short arm of chromosome 6A harbors the *Sr8* alleles, *Sr8a* and *Sr8b* (McIntosh 1972; Singh and McIntosh 1986), neither of which confers resistance to race TTKST. A gene in line SD4279 presumed to be *Sr8a* was recently mapped using the 9K SNP chip (Guerrero-Chavez *et al.* 2015), while another allele described as *Sr_TRTTF* was mapped in the Canadian wheat cultivar Harvest (Hiebert *et al.* 2017). The SNP markers *IWB64918* (*RFL_contig5170_330*) and *IWB6327* (*BS00011010_51*), which were linked to *Sr8155B1* in our study, were also reported by Hiebert *et al.* (2017) to be linked to *Sr_TRTTF*, which was predicted to be *Sr8a*. *IWB64918*, closely linked to *Sr8155B1*, mapped 3.3 cM from *Sr_TRTTF*, and *IWB6327* mapped 30.2 cM away from *Sr_TRTTF* (Hiebert *et al.* 2017). Allelism tests are needed to confirm the relationship between *Sr8155B1* and *Sr8*. Even though our data do not elucidate whether or not resistance in 8155-B1 is conferred by an allele at the *Sr8* locus, our phenotypic data do indicate that *Sr8155B1* is distinct from both *Sr8a* and *Sr8b*. Therefore, *Sr8155B1* is either a new allele at the *Sr8* locus or a new stem rust resistance gene.

The SNP marker *KASP 6AS_IWB10558* not only cosegregated with *Sr8155B1* in both populations derived from 8155-B1, but also predicted the presence/absence of this gene in unknown cultivars (Table 4). The robustness of this test would have been improved by including additional cultivars, especially susceptible cultivars. The KASP assay-based SNP marker developed for *Sr8155B1* in this study could be used in selecting for stem rust resistance in combination with other Ug99 resistance genes in durum wheat, such as *Sr13*. We deposited a hexaploid line from the Choteau/Mountrail population that possesses both *Sr13* and *Sr8155B1*. Hexaploid line “SXD 43,” deposited as PI 681713, was selected based on having gluten strength similar to Choteau, as well as solid stems related to wheat stem sawfly (*Cephus cinctus* Nort.) resistance inherited from Choteau (Lanning *et al.* 2004). SXD 43 could be used as a source of both *Sr8155B1* and *Sr13* for breeding common wheat varieties with resistance to race TTKST.

Races TTKST, TMLKC, TTKTT, and TTTSK exhibited low infection types of “0;” to “0;1” on 8155-B1, suggesting that the same gene in this monogenic line conditioned resistance against all these races. Although *Sr8155B1* confers susceptibility to race TTKSK, it conferred resistance to the three other races of the Ug99 race group tested. The specificity of *Sr8155B1* has implications for field stem rust screening in Africa. The international stem rust screening nursery in Njoro, Kenya, has been dominated by *Sr8155B1*-avirulent races TTKST and TTKTT (Newcomb *et al.* 2016), whereas the screening nursery in Debre Zeit, Ethiopia, has been dominated by the *Sr8155B1*-virulent race TTKSK, in addition to the presence of race JRCQC that is virulent to *Sr9e*, *Sr13* (Olivera *et al.* 2012), and *Sr8155B1* (Table 3). Gene *Sr9e* was thought to be common in North American durum (Klindworth *et al.* 2007; Olivera *et al.* 2012). It is possible that resistance postulated as *Sr9e* is, in fact, conferred by *Sr8155B1*, although further studies are needed to test this hypothesis. The observation that durum lines from North America that were resistant in Njoro became susceptible when tested in Debre Zeit (Olivera *et al.* 2012) may be a result of the presence of virulence to both *Sr13* and *Sr8155B1* in Debre Zeit.

The susceptibility of 8155-B1 to race TTKSK limits the value of *Sr8155B1* in protecting wheat cultivars from the Ug99 race group. However, the value of *Sr8155B1* lies in the high frequency of this gene in durum cultivars adapted to the Northern Great Plains of North America. For example, we postulated both Divide and Alkabo to possess *Sr8155B1*, and these two cultivars were the most widely planted durum varieties in North Dakota in 2015 and the first and third most widely planted durum varieties in North Dakota in 2016 (USDA National Agricultural Statistics Service). Going forward, we recommend the combination of both *Sr8155B1* and *Sr13*, such as in durum varieties Alkabo, Grenora, Mountrail, Munich, Renville, and Rugby in order to provide the maximum immediate protection of the durum crop in the Northern Great Plains of North America.

## 

## Supplementary Material

Supplemental material is available online at www.g3journal.org/lookup/suppl/doi:10.1534/g3.117.300209/-/DC1.
