# Supplementary figures and images for: Discovery of a Novel Stem Rust Resistance Allele in Durum Wheat that Exhibits Differential Reactions to Ug99 Isolates
Source: G3 (Bethesda). 2017 Aug 28;7(10):3481–90. doi: 10.1534/g3.117.300209 (PMC5633396; doi:10.1534/g3.117.300209)

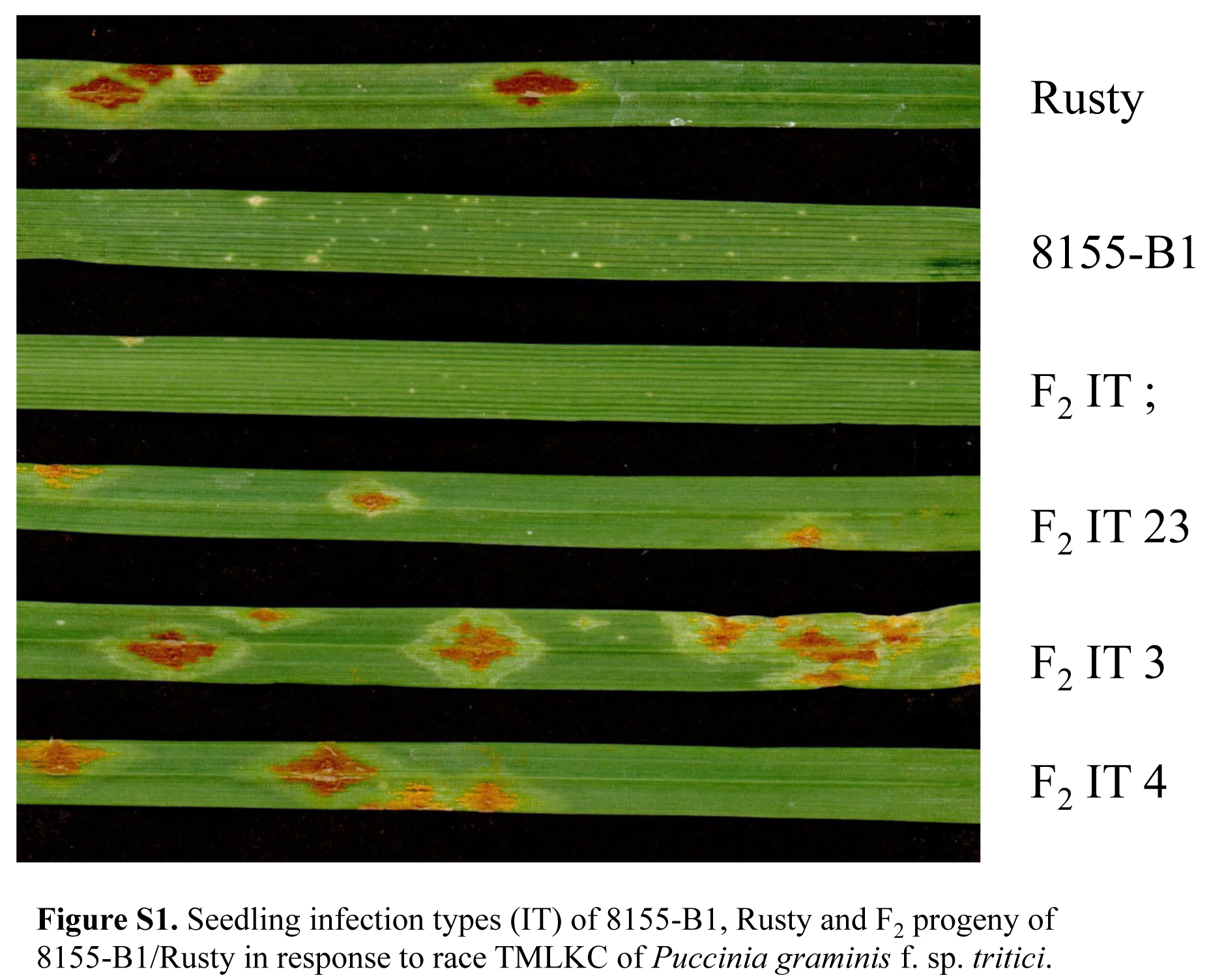

Supplement: Supplementary file 1 [file 3481FigureS1.tif]

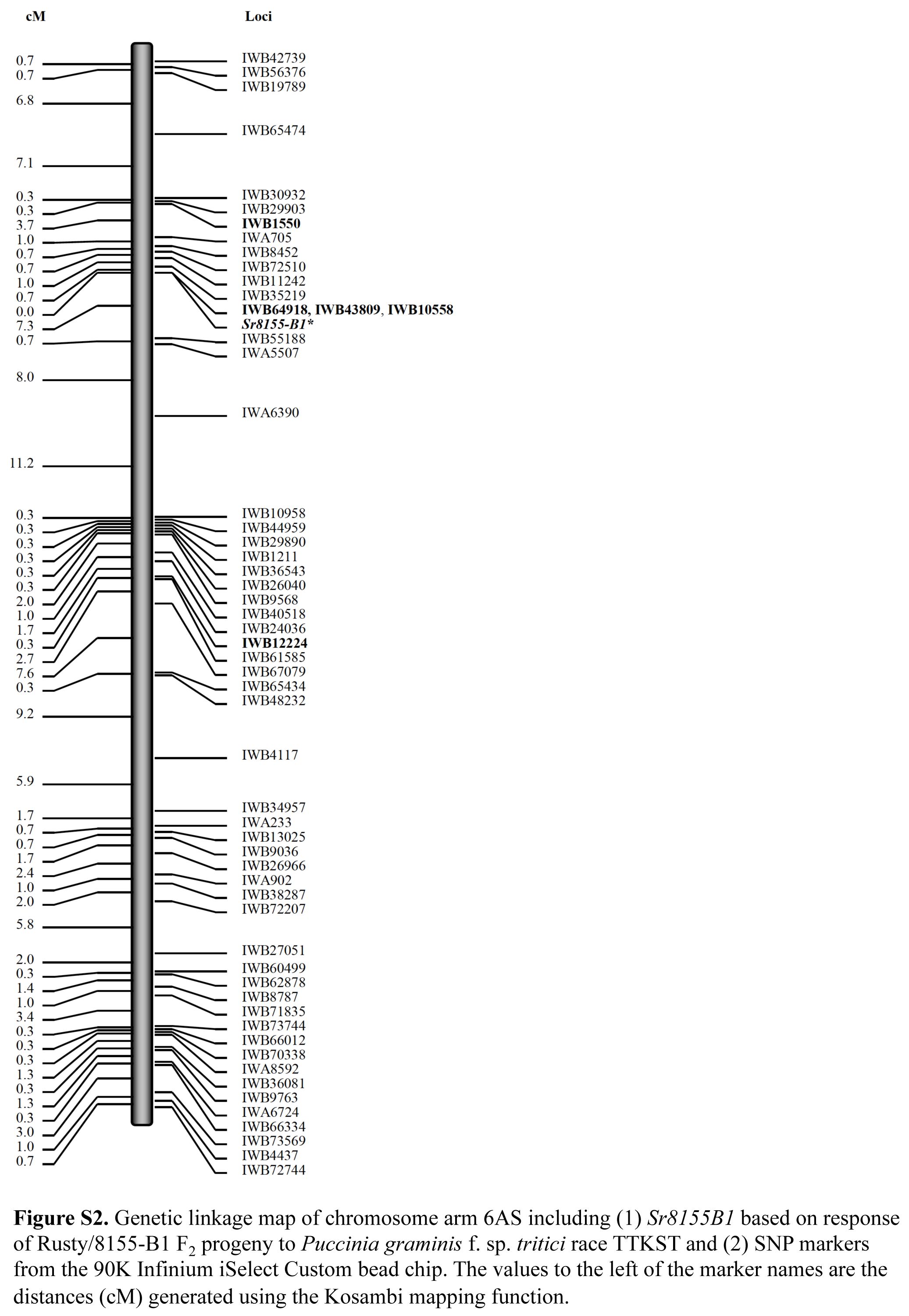

Supplement: Supplementary file 2 [file 3481FigureS2.tif]

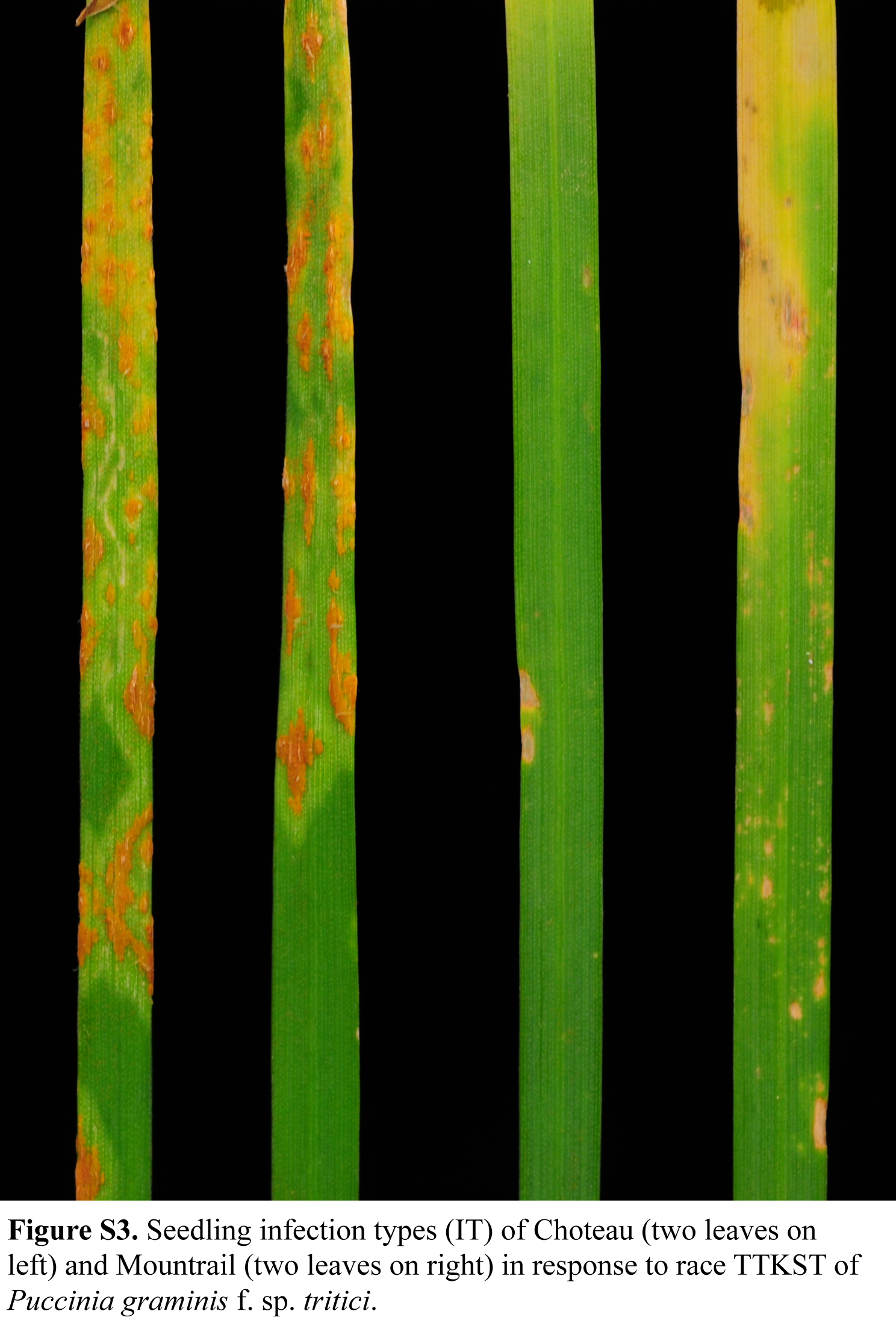

Supplement: Supplementary file 3 [file 3481FigureS3.tif]
